# Chemo Markers as Biomarkers in Septic Shock: A Comprehensive Review of Their Utility and Clinical Applications

**DOI:** 10.7759/cureus.42558

**Published:** 2023-07-27

**Authors:** Pulivarthi Chaithanya, Revat J Meshram

**Affiliations:** 1 Pediatrics, Jawaharlal Nehru Medical College, Datta Meghe Institute of Higher Education and Research, Wardha, IND; 2 Pediatrics, Jawaharlal Nehru Medical College, Datta Meghe Institute of Medical Sciences, Wardha, IND

**Keywords:** clinical applications, utility, septic shock, biomarkers, chemo markers

## Abstract

Sepsis is a life-threatening condition characterized by a dysregulated host response to infection, often leading to septic shock. Early diagnosis and prompt intervention are crucial for improving patient outcomes. Chemo markers, which are measurable biological substances associated with the pathophysiology of septic shock, have emerged as potential biomarkers for the identification, risk stratification, and management of this condition. This comprehensive review aims to thoroughly evaluate the utility and clinical applications of chemo markers in septic shock. The review begins by discussing the criteria for ideal chemo markers, including specificity, sensitivity, dynamic range, stability, non-invasiveness, and prognostic value. These characteristics ensure accurate diagnosis, early detection, effective monitoring, and prediction of clinical outcomes. Furthermore, the review explores the role of chemo markers in monitoring treatment response and disease progression, highlighting their ability to serve as objective indicators for assessing the effectiveness of interventions and making timely adjustments in management strategies. Moreover, the prognostic value of chemo markers in predicting outcomes is discussed, emphasizing their association with mortality, hospital stays, and the development of complications. Integration of chemo markers into prognostic models or scoring systems enhances risk stratification and informs therapeutic decisions. The review also delves into recent advances in chemo marker research and technology, emphasizing the potential for discovering novel chemo markers with enhanced diagnostic and prognostic capabilities. It highlights the use of high-throughput proteomics, genomics, and transcriptomics in identifying specific molecular signatures associated with septic shock. This contributes to a deeper understanding of the complex immune and inflammatory responses involved. In conclusion, chemo markers have emerged as valuable biomarkers in septic shock, offering potential utility in diagnosis, risk stratification, treatment monitoring, and prediction of outcomes. Continued research, validation, and integration into clinical practice are necessary to fully realize their potential in improving patient care and outcomes in septic shock.

## Introduction and background

Septic shock is a life-threatening condition characterized by a dysregulated immune response to infection, leading to widespread tissue damage and organ dysfunction. It remains a significant global health challenge, with high mortality rates despite advances in critical care management. Early diagnosis and accurate prognosis are crucial for initiating appropriate interventions and optimizing patient outcomes [1-3]. Septic shock arises from a cascade of complex pathophysiological events triggered by an infection, often caused by bacteria, viruses, or fungi. The initial infection releases pathogen-associated molecular patterns (PAMPs) and damage-associated molecular patterns (DAMPs), which activate the immune system. However, this immune response becomes dysregulated in septic shock, resulting in excessive inflammation, endothelial dysfunction, microcirculatory impairment, and multi-organ failure [3,4].

Early diagnosis of septic shock is critical as it allows for prompt initiation of appropriate therapies such as broad-spectrum antibiotics, fluid resuscitation, and vasopressors. Timely intervention within the golden hour significantly impacts patient survival and reduces the risk of long-term complications [5,6]. Prognosis in septic shock is challenging due to the heterogeneity of the disease and variations in patient response to treatment. Identifying patients at high risk for poor outcomes can guide clinical decision-making, resource allocation, and potential enrollment in targeted clinical trials [7,8]. Biomarkers play a crucial role in the diagnosis, risk stratification, and monitoring of septic shock. They provide objective measurements of biological processes or responses that can aid in identifying and characterizing the disease. Biomarkers can help differentiate sepsis from other conditions with similar clinical presentations, provide insights into the severity of infection, guide therapeutic decisions, and monitor response to treatment [9].

Chemo markers, also known as chemokines or chemotactic cytokines, are a subgroup of biomarkers that have garnered considerable attention in septic shock research. These small proteins are vital in immune cell recruitment, activation, and migration to the infection or tissue damage site. Given their involvement in the pathophysiology of septic shock, chemo markers have emerged as promising candidates for diagnostic and prognostic purposes in this condition [10,11]. This review article aims to comprehensively analyze the utility and clinical applications of chemo markers as biomarkers in septic shock. We aim to explore the current knowledge regarding specific chemo markers studied in septic shock, their diagnostic accuracy, prognostic value, and potential for guiding personalized treatment strategies. By synthesizing the available evidence, we highlight the significance of chemo markers in improving the early diagnosis, prognostication, and management of septic shock.

## Review

Methodology

**Figure 1:**
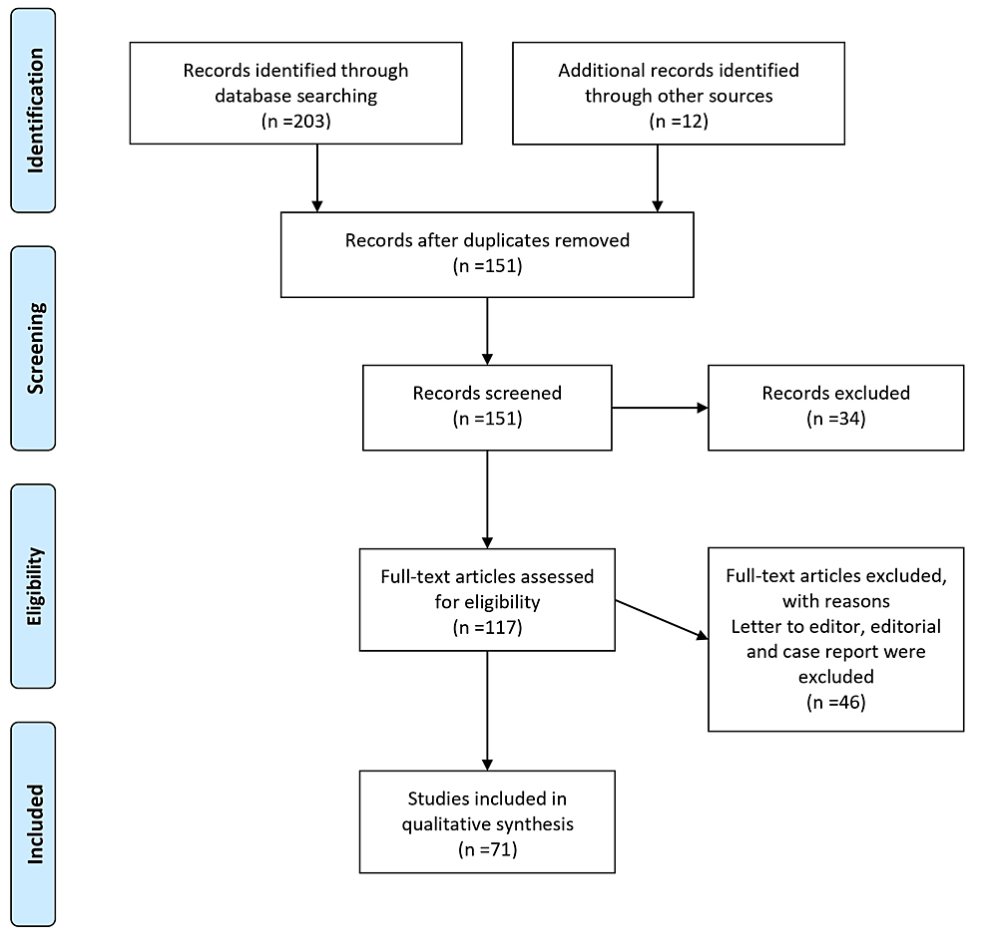
The selection process of articles used in this study. Adopted from the Preferred Reporting Items for Systematic Reviews and Meta-Analyses (PRISMA).

An extensive literature search was performed utilizing the PubMed, Scopus, and Embase databases to conduct this comprehensive review. The search strategy involved combining pertinent keywords and MeSH terms, including "chemo markers," "biomarkers," and "septic shock." The search was restricted to articles published in English from 2000 to 2023. No limitations were imposed regarding study design, publication type, or geographic location. A skilled medical librarian assisted in ensuring the search strategy's comprehensiveness. The selection process consisted of screening titles and abstracts and thoroughly evaluating full-text articles. Inclusion criteria encompassed clinical trials, observational studies, and systematic reviews focused on septic shock chemo markers. Additionally, animal studies about septic shock models were also considered. The review primarily focused on the diagnostic accuracy, prognostic value, and monitoring of treatment response associated with chemo markers. Exclusion criteria were applied to studies with insufficient data, inadequate sample sizes, or not directly addressing chemo markers in septic shock. Two reviewers independently conducted the screening and selection process, and any discrepancies that arose were resolved through discussion or consultation with a third reviewer when necessary. Figure 1 describes the selection process of articles used in our study.

Chemo markers: definition and characteristics

Definition of Chemo Markers

Chemo markers, also known as chemokines or chemotactic cytokines, are small signaling proteins that play a pivotal role in the immune response and inflammatory processes. They regulate the migration and activation of immune cells by binding to specific receptors on the cell surface. Chemo markers recruit immune cells to the site of infection or tissue damage, orchestrate their movement and positioning within tissues, and mediate communication between different cell types [12].

Types and Categories of Chemo Markers

CXC chemokines: This family of chemo markers is characterized by a conserved cysteine motif separated by an amino acid (X) and plays a significant role in neutrophil recruitment and activation. Examples of CXC chemokines include interleukin-8 (IL-8/CXCL8), growth-regulated oncogene (GRO/CXCL1, CXCL2, CXCL3), and epithelial-derived neutrophil-activating peptide 78 (ENA-78/CXCL5) [13,14].

CC chemokines: CC chemo markers contain adjacent cysteine residues and regulate monocyte, lymphocyte, and eosinophil recruitment. Monocyte chemoattractant protein-1 (MCP-1/CCL2), regulated upon activation, normal T cell expressed and secreted (RANTES/CCL5), and macrophage inflammatory protein-1 alpha (MIP-1α/CCL3) are examples of CC chemokines [15].

CX3C chemokines: This family includes a single member, fractalkine (CX3CL1), which is membrane bound and soluble. CX3CL1 is unique as it can act both as a chemoattractant for immune cells and as an adhesion molecule, mediating cell-cell interactions [16,17].

Characteristics of Ideal Chemo Markers for Septic Shock

Specificity: Chemo markers in septic shock should exhibit specificity to this condition, allowing differentiation from other causes of systemic inflammation. This specificity ensures accurate diagnosis by minimizing false positives or misinterpretation of results. By specifically indicating septic shock, these markers enable clinicians to distinguish it from similar conditions and provide targeted interventions [18,19].

Sensitivity: Chemo markers should demonstrate high sensitivity, enabling the detection of septic shock in its early stages or in patients with atypical presentations. Early detection is crucial as septic shock can progress rapidly and lead to severe organ dysfunction or mortality. Highly sensitive chemo markers facilitate timely intervention, allowing healthcare providers to initiate appropriate treatment promptly, which can significantly improve patient outcomes [20,21].

Dynamic range: Chemo markers in septic shock should possess a broad dynamic range, meaning that they can detect and quantify the varying stages of the disease. This characteristic allows for the discrimination between different disease severities and provides insights into the progression and response to treatment. By capturing the dynamic changes in chemo marker levels, clinicians can monitor the effectiveness of interventions and tailor treatment strategies accordingly [22,23].

Stability: To ensure reliable measurement and storage, ideal chemo markers should demonstrate stability in various clinical samples, such as blood or urine. Stability is essential for the standardization and reproducibility of results, allowing for accurate comparisons across different studies and clinical settings. Stable chemo markers can be measured reliably over time, facilitating longitudinal monitoring of patients and assessment of treatment response [24,25].

Non-invasiveness: Chemo markers that can be measured through non-invasive sampling methods, such as blood or urine, are preferable. Non-invasive sampling reduces patient discomfort and complications associated with invasive procedures. It also enables repeated measurements, which are often necessary to monitor the progression of septic shock or evaluate treatment efficacy. Chemo markers that can be easily measured through non-invasive means enhance the feasibility of their use in routine clinical practice [26,27].

Prognostic value: Chemo markers should possess prognostic value, meaning that they correlate with clinical outcomes such as mortality, length of hospital stays, or development of complications in septic shock patients. Chemo markers with prognostic value aid in risk stratification, allowing healthcare providers to identify high-risk patients who may require more intensive management or closer monitoring. Additionally, prognostic chemo markers can guide therapeutic decisions and help clinicians make informed choices regarding treatment options for better patient outcomes [28,29]. Understanding these characteristics is crucial for evaluating the potential utility of chemo markers as biomarkers in septic shock. In the subsequent sections, we will explore specific chemo markers studied in septic shock, their diagnostic accuracy, and their role in guiding clinical management.

Utility of chemo markers in septic shock diagnosis

Role of Chemo Markers in Early Detection and Diagnosis of Septic Shock

Chemo markers play a crucial role in the early detection and diagnosis of septic shock. The dysregulated immune response in septic shock leads to the release of chemo markers, which can be measured in clinical samples such as blood or urine. By assessing the levels of specific chemo markers, healthcare providers can identify patients at risk of or in the early stages of septic shock, initiating appropriate interventions promptly [30,31].

Chemo markers provide valuable information regarding the presence and severity of systemic inflammation, aiding in the differentiation of septic shock from other conditions with similar clinical presentations. The ability to detect septic shock early allows for the timely administration of antibiotics, resuscitation fluids, and vasopressors, which is crucial for improving patient outcomes [32].

Comparative Analysis of Chemo Markers With Other Biomarkers

Compared to other biomarkers used in septic shock, chemo markers offer several advantages. They are directly involved in the immune response and inflammatory processes specific to septic shock, making them more specific to this condition than general inflammatory markers. Chemo markers also have the potential to provide insights into the ongoing immune response and the dynamic changes occurring during septic shock [33,34].

Compared to traditional biomarkers such as C-reactive protein (CRP) and procalcitonin (PCT), chemo markers offer a more dynamic assessment of the immune response. Chemo markers reflect the activation and migration of immune cells, providing a more nuanced understanding of the inflammatory process. This dynamic nature allows for monitoring the response to treatment and adjusting therapeutic strategies accordingly [35,36].

Diagnostic Accuracy and Predictive Value of Chemo Markers

Chemo markers have demonstrated varying diagnostic accuracy and predictive value in septic shock. Several studies have reported their association with disease severity and clinical outcomes, including mortality, length of hospital stay, and development of organ dysfunction [37]. Diagnostic accuracy depends on the specific chemo marker being investigated. Some chemo markers may have higher sensitivity or specificity in detecting septic shock than others. Combining multiple chemo markers or incorporating them into predictive models may enhance diagnostic accuracy [38].

Furthermore, the predictive value of chemo markers extends beyond diagnosis. Serial measurements of chemo markers can provide valuable information regarding treatment response and prognostication. Changes in chemo marker levels over time can guide clinicians in assessing the effectiveness of interventions and predicting clinical outcomes [39,40].

Challenges and Limitations in the Clinical Implementation of Chemo Markers

Despite their potential utility, there are challenges and limitations to consider in the clinical implementation of chemo markers in septic shock diagnosis. Some chemo markers lack specificity to septic shock and can be elevated in other inflammatory conditions, leading to false-positive results. Differentiating septic shock from other causes of systemic inflammation remains challenging [41].

Furthermore, the availability and standardization of chemo marker assays may vary, leading to measurement discrepancies across different laboratories. Variations in sample collection, processing, and storage can also impact the reliability and comparability of results. Addressing these challenges and ensuring consistent and accurate measurement of chemo markers are important for their successful integration into routine clinical practice [42,43]. Additionally, chemo marker levels should be interpreted in the context of clinical presentation, patient characteristics, and other diagnostic information. Chemo markers should not be used in isolation but as part of a comprehensive diagnostic and prognostic approach.

Clinical applications of chemo markers in septic shock management

Monitoring Response to Treatment and Disease Progression

Monitoring the response to treatment and disease progression in septic shock is a critical aspect of patient management, and chemo markers can provide valuable insights in this regard. By serially measuring chemo marker levels, clinicians can objectively assess the effectiveness of interventions and make informed decisions regarding treatment adjustments [44,45].

A decrease in chemo marker levels over time can indicate a favorable response to treatment. It suggests that the therapeutic interventions are addressing the underlying inflammatory processes associated with septic shock and leading to a reduction in chemo marker release. Monitoring the decline in chemo marker levels can provide reassurance that the treatment has the desired effect and guide clinicians in continuing the current therapeutic regimen [46,47].

On the other hand, persistently elevated chemo marker levels or an upward trend in their measurements may suggest an inadequate response to treatment or disease progression. This scenario alerts clinicians to the possibility that the ongoing interventions are not effectively controlling the inflammatory response or that the condition is worsening despite treatment efforts. In such cases, it is crucial to reevaluate the treatment plan and consider alternative strategies to optimize patient outcomes [48].

Serial measurements of chemo markers also enable the identification of rapid changes in disease progression. A sudden and significant increase in chemo marker levels may indicate a deteriorating condition or the onset of complications, prompting immediate action. By closely monitoring chemo marker trends, clinicians can promptly recognize and respond to these changes, implementing appropriate interventions to mitigate further deterioration and improve patient outcomes [49].

It is important to note that chemo markers should not be relied upon as the sole factor for treatment decision-making. They should be interpreted in conjunction with other clinical parameters and patient-specific factors. Nevertheless, chemo markers provide objective and quantifiable information that can aid in monitoring the response to treatment and disease progression in septic shock. Regular measurements and careful analysis of chemo marker trends contribute to a comprehensive assessment of a patient's clinical status, guiding therapeutic decisions and facilitating the delivery of personalized care [50].

Prognostic Value of Chemo Markers in Predicting Outcomes

Chemo markers in septic shock have demonstrated prognostic value, providing valuable information about the likely clinical outcomes for affected individuals. Elevated levels of specific chemo markers have been consistently associated with worse prognosis in septic shock patients [30].

One important clinical outcome that chemo markers can predict is mortality. Several studies have identified certain chemo markers whose elevated levels are associated with increased mortality rates in septic shock. These markers reflect the underlying inflammatory response's severity and the organ dysfunction's extent. By incorporating these chemo markers into prognostic models or scoring systems, clinicians can improve risk stratification and identify patients at higher mortality risk. This information enables healthcare providers to allocate appropriate resources, implement more intensive management strategies, and make informed decisions regarding end-of-life care [18,29,31,33].

Prolonged hospital stays are another significant outcome that can be predicted using chemo markers. Higher levels of specific chemo markers have been linked to extended periods of hospitalization in septic shock patients. These markers reflect the severity of the systemic inflammatory response and the complexity of the disease. By considering chemo marker measurements in conjunction with other clinical factors, clinicians can better anticipate the duration of hospitalization and allocate appropriate resources accordingly [51].

In addition to mortality and hospital stays, elevated chemo marker levels have been associated with developing organ dysfunction in septic shock patients. Certain chemo markers indicate ongoing cellular damage and dysfunction in vital organs, such as the liver, kidneys, or cardiovascular system. By monitoring these markers, clinicians can identify patients at higher risk of developing organ dysfunction and initiate targeted interventions to mitigate further deterioration [52].

By incorporating chemo marker measurements into prognostic models or scoring systems, clinicians can enhance risk stratification and obtain a more comprehensive understanding of a patient's prognosis. This information enables healthcare providers to tailor treatment plans, allocate resources effectively, and provide appropriate support and counseling to patients and their families [53]. It is important to note that while chemo markers provide valuable prognostic information, they should be interpreted in conjunction with other clinical parameters and patient-specific factors. Prognostication in septic shock is complex, and multiple variables contribute to the overall prognosis. Chemo markers are additional tools that enhance prognostic accuracy and assist clinicians in delivering personalized care to septic shock patients [54].

Guiding Personalized Treatment Strategies Based on Chemo Marker Profiles

Chemo marker profiles can potentially guide personalized treatment strategies in septic shock. Different patterns of chemo marker expression may reflect distinct immunological subtypes or phenotypes, suggesting heterogeneous underlying mechanisms and responses to therapy. Tailoring treatment based on individual chemo marker profiles may help optimize patient outcomes by identifying patients who may benefit from specific therapies or targeted interventions [55].

For example, patients with a chemo marker profile indicating excessive inflammation may benefit from immunomodulatory therapies. In contrast, those with a profile suggesting impaired immune response may require interventions to enhance host defense. Chemo markers can help identify patients who may benefit from specific therapeutic approaches, such as anti-inflammatory agents, immune modulators, or antimicrobial agents tailored to pathogen profiles [56].

Incorporation of Chemo Markers in Sepsis Guidelines and Protocols

The increasing evidence supporting the clinical utility of chemo markers in septic shock paves the way for their potential incorporation into sepsis guidelines and protocols. By including specific chemo markers in these guidelines, healthcare providers can benefit from standardized approaches for utilizing chemo markers in clinical practice. This integration would facilitate the systematic assessment of septic shock, leading to early recognition, appropriate interventions, and consistent monitoring of patients [57].

The inclusion of chemo markers in sepsis guidelines and protocols has several advantages. First, it promotes the widespread adoption and utilization of chemo markers in clinical practice. Healthcare providers who follow the guidelines are more likely to incorporate chemo marker testing into their routine assessment of septic shock patients, leading to increased awareness and utilization of these valuable tools [53]. Second, incorporating chemo markers in guidelines and protocols fosters a multidisciplinary approach to septic shock management. Chemo markers are typically measured in the laboratory setting, involving collaboration between clinicians, laboratory staff, and other healthcare professionals. Incorporating chemo markers into guidelines emphasizes the importance of multidisciplinary teamwork and encourages effective communication and coordination among different healthcare disciplines [58]. Third, integrating chemo markers in guidelines and protocols can help standardize the interpretation of results and guide clinicians in decision-making. Specific cutoff values or trends in chemo marker levels can be included in the guidelines to aid in the diagnosis, risk stratification, and treatment monitoring. This standardized approach reduces variability in the interpretation of chemo marker results, ensuring consistent practices and improving the quality of patient care [59].

However, it is important to recognize that incorporating chemo markers into sepsis guidelines and protocols requires further research, validation, and consensus building. The selection of specific chemo markers, determination of appropriate cutoff values, and consideration of the strengths and limitations of different markers need to be carefully evaluated. Additionally, the feasibility, cost-effectiveness, and accessibility of chemo marker testing should be considered [25]. Collaboration among researchers, clinicians, guideline committees, and professional societies is essential to establish robust guidelines and protocols that integrate chemo markers effectively and appropriately. These guidelines should be regularly updated to reflect advances in chemo marker research and evolving clinical evidence.

Future directions and emerging trends

Advances in Chemo Marker Research and Technology

The chemo marker research in septic shock is rapidly evolving, driven by ongoing technological advancements and a growing understanding of the underlying pathophysiology. Continued research efforts are expected to yield significant advancements in discovering, validating, and utilizing chemo markers in septic shock. Here are some key areas of progress:

Discovery of novel chemo markers: Continued research will likely lead to identifying novel chemo markers with enhanced diagnostic and prognostic capabilities in septic shock. Through comprehensive analysis of chemo marker profiles using advanced technologies, researchers can uncover specific molecular signatures associated with septic shock. These signatures may provide valuable insights into the complex immune and inflammatory responses underlying the condition [60].

Technological advancements: Advancements in technology, such as high-throughput proteomics, genomics, and transcriptomics, will greatly enhance chemo marker research in septic shock. These technologies allow for the simultaneous analysis of multiple chemo markers and their corresponding molecular pathways on a large scale. By applying these high-throughput approaches, researchers can uncover previously unrecognized chemo markers and elucidate their roles in septic shock pathogenesis and patient outcomes [61].

Integration of multi-omics approaches: Integration of data from various omics disciplines, including genomics, proteomics, transcriptomics, and metabolomics, holds great promise in advancing chemo marker research. Integrative multi-omics analyses provide a comprehensive understanding of the molecular mechanisms involved in septic shock, enabling the identification of robust chemo marker signatures associated with the condition. Such approaches can contribute to developing more accurate diagnostic and prognostic models [62].

Biomarker panels and personalized medicine: As research progresses, the focus will shift toward developing biomarker panels comprising multiple chemo markers with complementary roles in septic shock diagnosis, prognosis, and treatment response. Combining multiple markers may improve diagnostic accuracy and provide a more comprehensive disease severity and progression assessment. Additionally, the incorporation of chemo marker data into personalized medicine approaches has the potential to guide individualized treatment strategies, improving patient outcomes and reducing the burden of septic shock [63].

Potential Novel Chemo Markers Under Investigation

Immune cell subset markers: Immune cell subsets play a crucial role in the immune response during septic shock. Researchers are exploring the use of chemo markers associated with specific immune cell populations, such as monocytes, neutrophils, or T cells. These markers may provide valuable information about the activation status, functional phenotypes, and dysregulation of immune cells in septic shock, allowing for a more comprehensive understanding of the immune response and its impact on patient outcomes [64].

MicroRNAs: MicroRNAs (miRNAs) are small non-coding RNA molecules that regulate gene expression. They have emerged as potential chemo markers due to their stability in various clinical samples, involvement in immune regulation, and ability to reflect pathological processes. Ongoing research aims to identify specific miRNAs that are dysregulated in septic shock and correlate with disease severity or clinical outcomes. Exploring the role of miRNAs as chemo markers may provide insights into the molecular mechanisms underlying septic shock and offer novel diagnostic or prognostic tools [65].

Cytokines and inflammatory mediators: Cytokines and other inflammatory mediators are critical components of the immune response in septic shock. While some well-known chemo markers in this category, such as interleukin-6 (IL-6) and C-reactive protein (CRP), have been extensively studied, ongoing research seeks to identify additional cytokines or mediators that can provide complementary information. Novel chemo markers in this group may include less commonly studied cytokines, chemokines, or signaling molecules involved in the intricate regulation of inflammation during septic shock [66].

Metabolites: Metabolomic profiling is an emerging field investigating the comprehensive set of small-molecule metabolites in biological samples. Researchers are exploring the potential of metabolites as chemo markers in septic shock. Metabolomic analysis can reveal alterations in metabolic pathways associated with septic shock, highlighting the impact of metabolic dysregulation on disease pathogenesis and progression. Identifying specific metabolites associated with septic shock may provide insights into novel therapeutic targets or serve as markers for monitoring treatment response [67].

Integration of Chemo Markers into Point-of-Care Testing and Precision Medicine

Point-of-care testing: Rapid and reliable point-of-care testing platforms for chemo markers can provide immediate results at the patient's bedside, eliminating the need for time-consuming laboratory analysis. This real-time assessment of chemo marker levels enables clinicians to make prompt and informed decisions regarding patient management. Point-of-care testing facilitates early detection of septic shock, enables timely initiation of treatment, and allows for close monitoring of chemo marker dynamics throughout the disease. By integrating chemo markers into point-of-care testing, healthcare providers can enhance the speed and accuracy of diagnosis, risk stratification, and treatment monitoring, ultimately improving patient outcomes [68].

Precision medicine: Precision medicine approaches consider individual patient characteristics, such as genetic variations, chemo marker profiles, and other relevant clinical variables, to guide personalized treatment decisions. Incorporating chemo markers into precision medicine strategies enables risk stratification and treatment selection based on an individual's specific chemo marker profile. By considering each patient's unique chemo marker signature, healthcare providers can tailor interventions to address the underlying pathophysiology and optimize therapeutic efficacy. Precision medicine approaches may also help identify patients more likely to benefit from certain interventions or require more aggressive management strategies. This individualized approach enhances the potential for positive clinical outcomes and minimizes the risk of unnecessary treatments or adverse effects [69].

Data integration and decision support: Integrating chemo marker data into electronic health records (EHRs) and clinical decision support systems can facilitate comprehensive data analysis and interpretation. By integrating chemo marker results with other clinical parameters and patient data, healthcare providers can obtain a holistic view of the patient's condition and make well-informed decisions. Decision support systems incorporating chemo marker data can provide evidence-based recommendations for treatment options, dosage adjustments, or additional diagnostic tests. Data integration enhances clinical decision-making, streamlines workflows, and promotes standardized and consistent use of chemo markers in septic shock management [70].

Implications for the Future Management of Septic Shock

The incorporation of chemo markers into routine clinical practice has the potential to revolutionize the management of septic shock. Chemo markers as diagnostic and prognostic tools can aid in early detection, risk stratification, and tailored therapeutic interventions. This, in turn, may lead to improved patient outcomes, reduced healthcare costs, and optimized resource utilization [71].

Furthermore, integrating chemo markers into septic shock management algorithms and guidelines may enhance standardization and consistency in clinical practice. It may also foster collaboration among healthcare professionals from various specialties, promoting a multidisciplinary approach to septic shock management. Overall, the future of chemo markers in septic shock management holds great promise. Continued research, technological advancements, and integration into clinical practice are expected to refine our understanding of septic shock pathophysiology, enhance diagnostic accuracy, and guide personalized therapeutic strategies, ultimately improving patient outcomes in this life-threatening condition.

## Conclusions

Further research and clinical validation are crucial to establish chemo markers' robustness and clinical utility in septic shock. More studies are needed to validate specific chemo markers' diagnostic and prognostic accuracy and evaluate their performance in diverse patient populations. Standardization of assays, establishing appropriate cutoff values, and incorporating them into predictive models or scoring systems will enhance their clinical applicability. Additionally, understanding chemo markers' kinetics and temporal changes in septic shock is essential for accurate interpretation and clinical decision-making. Longitudinal studies and integration with other clinical and laboratory parameters can provide a more comprehensive understanding of their role in septic shock management. The integration of chemo markers into septic shock management has the potential to impact patient outcomes significantly. Early detection through chemo marker assessment can lead to timely initiating appropriate interventions, such as early administration of antibiotics and targeted therapies. Monitoring treatment response using chemo markers can guide therapy adjustments, ensuring optimized patient care. Prognostic information provided by chemo markers may assist in risk stratification and help identify patients who require more intensive monitoring or aggressive interventions. Furthermore, incorporating chemo markers into point-of-care testing and precision medicine approaches can facilitate individualized management strategies, leading to improved outcomes and personalized care for patients with septic shock.
